# Integrative radiomics and habitat imaging models for predicting PD-L1 expression in non-small cell lung cancer

**DOI:** 10.3389/fonc.2026.1786749

**Published:** 2026-07-06

**Authors:** Hao Fang, Huadong Chen, Wei Tan, Peijun Liu

**Affiliations:** 1 Department of Respiratory and Critical Care Medicine, The Central Hospital of Enshi Tujia and Miao Autonomous Prefecture, Enshi, China; 2 Department of Radiology, The Central Hospital of Enshi Tujia and Miao Autonomous Prefecture, Enshi, China

**Keywords:** habitat imaging, non-small cell lung cancer, PD-L1 expression, radiomics, tumor heterogeneity

## Abstract

**Objective:**

To evaluate the feasibility of an arterial-phase computed tomography (CT)–based radiomics and habitat imaging model for noninvasive prediction of programmed death-ligand 1 (PD-L1) expression in non–small cell lung cancer (NSCLC).

**Methods:**

This retrospective study included 246 patients with pathologically confirmed NSCLC who underwent arterial-phase CT between January 2021 and March 2024. Patients were randomly divided into a training cohort (n = 172) and a validation cohort (n = 74). Whole-tumor radiomics features were extracted from manually segmented lesions, while tumor habitats were generated using texture-based clustering to derive habitat imaging features. After feature selection, whole-tumor radiomics, habitat imaging, and combined models incorporating clinical variables were constructed using logistic regression. Model performance was assessed using the area under the receiver operating characteristic curve (AUC) and decision curve analysis.

**Results:**

Tumor maximum diameter and intratumoral necrosis were identified as independent clinical predictors of PD-L1 expression. The habitat imaging model outperformed the whole-tumor radiomics model, achieving AUCs of 0.774 and 0.758 in the training and validation cohorts, respectively. The combined model demonstrated the best performance, with AUCs of 0.840 in the training cohort and 0.828 in the validation cohort, and showed superior net clinical benefit on decision curve analysis.

**Conclusion:**

An arterial-phase CT–based habitat imaging model provides effective noninvasive prediction of PD-L1 expression in NSCLC. Integration of radiomics and clinical features further improves predictive performance, highlighting its potential utility for pre-immunotherapy evaluation.

## 1 Introduction

Non–small cell lung cancer (NSCLC) accounts for approximately 80–85% of all lung cancer cases and remains one of the leading causes of cancer-related mortality worldwide. Despite advances in diagnostic techniques and therapeutic strategies, the overall prognosis of patients with NSCLC remains unsatisfactory (1). Early detection and accurate stratification of patients are therefore essential for improving long-term survival outcomes (2). In recent years, increasing insights into the tumor immune microenvironment have highlighted the critical role of immune checkpoint inhibitors in the treatment of NSCLC (3, 4). Among these biomarkers, programmed death-ligand 1 (PD-L1) expression has emerged as an important predictor of response to immunotherapy, and patients with high PD-L1 expression have demonstrated meaningful clinical benefit from immune-based treatments (5, 6).

At present, assessment of PD-L1 expression relies predominantly on immunohistochemical analysis of tumor tissue (7, 8). However, this approach is invasive and subject to sampling bias caused by tumor heterogeneity, limited tissue volume, and variability in biopsy sites (9). In addition, pathological evaluation may not fully capture the spatial heterogeneity of the tumor microenvironment and is associated with inherent delays in clinical decision-making. Radiomics offers a noninvasive alternative by extracting high-dimensional quantitative features from routine medical images, enabling the characterization of intratumoral heterogeneity before treatment and providing complementary information for diagnosis, therapeutic evaluation, and prognostic assessment (10, 11). Extending this framework, habitat imaging decomposes tumors into multiple spatial compartments defined by distinct imaging patterns, offering a more nuanced representation of intratumoral heterogeneity and microenvironmental complexity.

Compared with non-contrast CT, contrast-enhanced CT provides additional information related to tumor perfusion and microcirculation, which may facilitate more accurate identification of tumor habitats and characterization of the tumor microenvironment (12, 13). In routine thoracic imaging, arterial-phase CT is one of the most consistently acquired contrast-enhanced phases across institutions and offers favorable reproducibility and clinical applicability (14). Therefore, arterial-phase CT was selected as the imaging basis for habitat analysis in this study to enhance model generalizability. Although radiomics-based approaches have been increasingly applied to molecular characterization and immunological biomarker prediction in NSCLC, studies focusing on habitat imaging for noninvasive prediction of PD-L1 expression remain limited (15). Accordingly, this study aimed to develop arterial-phase CT–based whole-tumor radiomics and habitat imaging models for predicting PD-L1 expression in NSCLC, providing imaging support for pre-immunotherapy assessment and individualized treatment planning.

## 2 Materials and methods

### 2.1 Study population

This retrospective study enrolled patients with pathologically confirmed NSCLC who underwent chest CT at Enshi Central Hospital between January 2021 and March 2024. A total of 246 eligible patients were included after screening. Histological subtypes comprised lung adenocarcinoma, lung squamous cell carcinoma, large cell carcinoma, and adenosquamous carcinoma. The inclusion criteria were as follows: (1) completion of non-contrast and arterial-phase contrast-enhanced chest CT before surgery or systemic treatment. (2) no prior antitumor therapy, including chemotherapy, radiotherapy, targeted therapy, or immunotherapy, before CT examination, and (3) availability of immunohistochemical results for PD-L1 expression. The exclusion criteria included: (1) history of other malignancies. (2) prior percutaneous biopsy or other invasive procedures before CT acquisition, and (3) poor image quality or indistinct tumor margins that could affect region-of-interest delineation and feature extraction. Eligible patients were randomly assigned to a training cohort (n = 172) and a validation cohort (n = 74) at a ratio of 7:3. This study was approved by the Ethics Committee of Enshi Central Hospital, and written informed consent was obtained from all participants.

### 2.2 Assessment of PD-L1 expression

PD-L1 expression was evaluated using a validated immunohistochemical staining assay performed on tumor tissue specimens. PD-L1 positivity was defined as membranous staining of tumor cells regardless of staining intensity, whereas cytoplasmic staining and staining of tumor-associated immune cells, such as macrophages or lymphocytes, were not considered. PD-L1 expression levels were categorized according to the proportion of PD-L1–positive tumor cells as follows: negative expression (<1%), low expression (1–49%), and high expression (≥50%) (13).

### 2.3 CT scanners and acquisition protocol

All imaging examinations were performed using multidetector CT scanners (GE Revolution CT and Canon Aquilion ONE). Scans were obtained from the lung apex to the lung base with patients in the supine position. The acquisition parameters were as follows: tube voltage of 100–120 kV, automatic tube current modulation, slice thickness of 1.0 mm, and a reconstruction interval of 1.0 mm. Images were reconstructed using both lung window settings (window width, 1,500 HU; window level, −600 HU) and mediastinal window settings (window width, 350 HU; window level, 50 HU). Following the non-contrast scan, a nonionic iodinated contrast agent (iopromide, 370 mg I/mL) was administered intravenously through the antecubital vein at a flow rate of 3.0 mL/s. The total contrast volume was weight-adjusted (1.0–1.2 mL/kg). Arterial-phase contrast-enhanced images were acquired with a fixed delay of 25 s after contrast injection.

### 2.4 Conventional imaging feature assessment

All CT images were independently reviewed by two radiologists with more than five years of experience in thoracic imaging, both blinded to pathological and immunohistochemical results. In cases of disagreement, a consensus was reached through joint discussion. The evaluated conventional imaging features included tumor location, maximum diameter, CT attenuation values on non-contrast and arterial-phase images, tumor morphology (lobulation and spiculation), pleural-related signs (pleural retraction and pleural invasion), intratumoral characteristics (necrosis, cavitation, and calcification), peritumoral findings (obstructive pneumonia, small-vessel convergence or penetration signs), presence of intrapulmonary metastases, mediastinal or hilar lymphadenopathy, and concomitant pleural or pericardial effusion.

### 2.5 Radiomics and habitat imaging feature extraction

**Figure 1:**
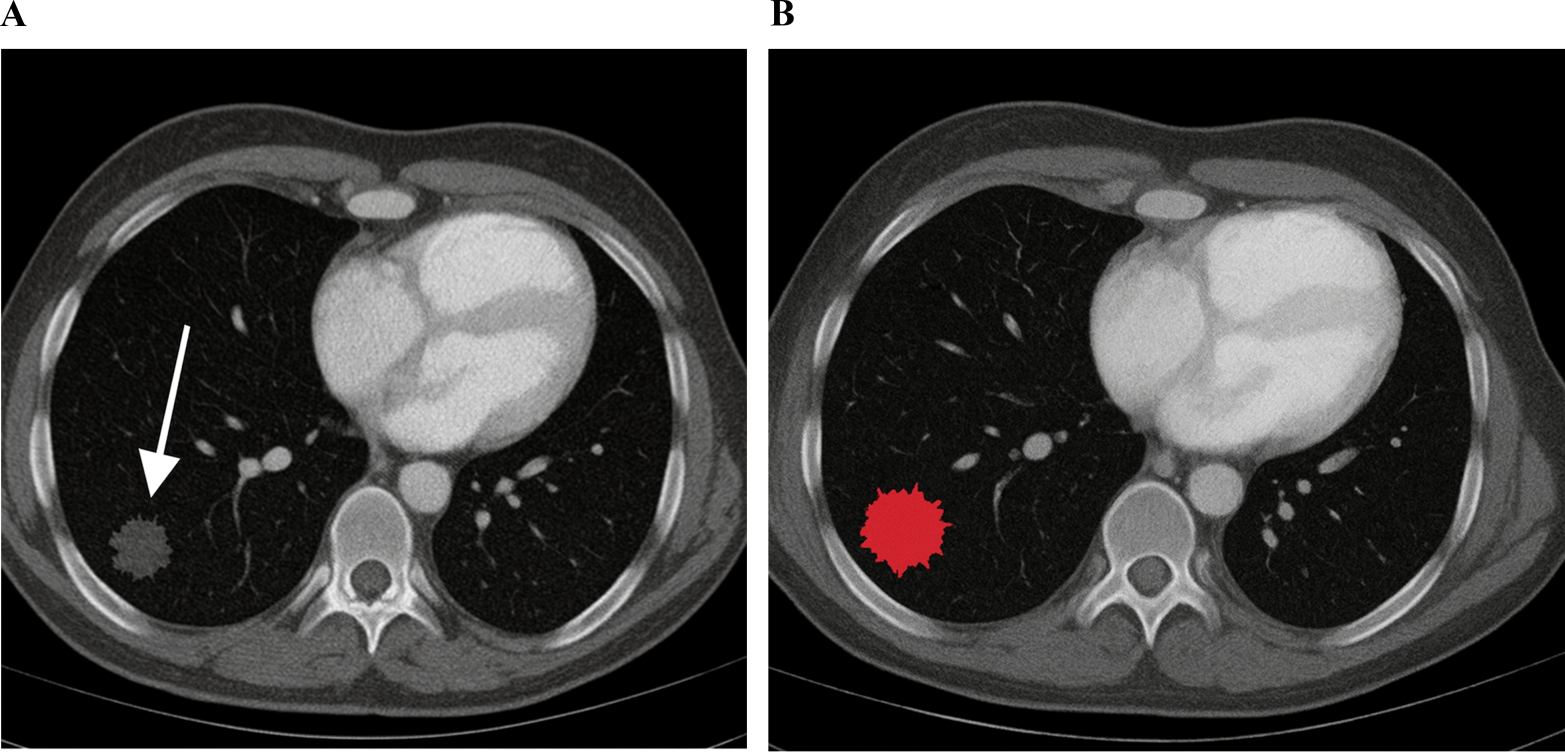
**(A)** A 69-year-old male patient with adenocarcinoma of the right lower lobe of the lung (arrow). **(B)** Tumor region of interest (ROI) (arrow).

Arterial-phase contrast-enhanced CT images of all enrolled patients were exported from the PACS in DICOM format and imported into ITK-SNAP software (version 3.8.0) for image processing. One radiologist, blinded to pathological findings and PD-L1 expression status, manually delineated the region of interest (ROI) slice by slice along the tumor boundary, carefully excluding adjacent vessels, bronchi, and normal lung parenchyma (**Figure 1**). To assess the reproducibility and reliability of ROI delineation and feature extraction, patients were randomly selected. After a one-month interval, ROI segmentation was repeated independently by Reader A and another radiologist (Reader B) using the same criteria. Intra-class correlation coefficients (ICC) were calculated to evaluate both inter-observer and intra-observer agreement. Only features with ICC values > 0.80 were retained for subsequent analysis. Prior to feature extraction, all CT images underwent standardized preprocessing to reduce variability associated with different scanners and acquisition parameters. Images were resampled to isotropic voxels using linear interpolation, and voxel intensities were normalized using Z-score normalization. Gray-level discretization was performed using a fixed bin-width strategy before texture feature extraction.

The finalized ROIs were subsequently imported into the PyRadiomics platform (version 3.1) for feature extraction. Radiomic features were extracted according to the recommendations of the Image Biomarker Standardization Initiative (IBSI). The extracted feature categories included first-order statistical features, shape features, gray-level co-occurrence matrix (GLCM) features, gray-level run-length matrix (GLRLM) features, gray-level size-zone matrix (GLSZM) features, gray-level dependence matrix (GLDM) features, neighboring gray-tone difference matrix (NGTDM) features, and wavelet-transformed features. A complete list of extracted radiomic features is provided in **Supplementary Table 1**. First, whole-tumor radiomics features were extracted. Subsequently, local radiomics features were obtained using a non-overlapping 3 × 3 × 3 sliding window centered on each voxel within the ROI. A total of 19 categories of local features were generated, including entropy and energy-related parameters. Based on these local features, unsupervised K-means clustering (K ranging from 2 to 15) was applied to identify spatial subregions within tumors that shared similar imaging characteristics. Clustering was performed using pooled data from all patients to ensure consistency and stability of habitat partitioning across individuals. Euclidean distance was used as the similarity metric, and the optimal number of clusters (n) was determined by maximizing the Calinski–Harabasz index. For visualization, voxels assigned to the same cluster were labeled with identical colors, thereby generating multiple intratumoral subregions with distinct spatial distributions. Radiomics features were then extracted separately from each subregion. Finally, subregional features were integrated using a K-nearest neighbor algorithm to construct the habitat imaging feature set, which was subsequently used for model development and analysis.

### 2.6 Model construction and evaluation

All whole-tumor radiomics features and habitat imaging features included in the analysis were normalized using Z-score standardization. In the training cohort, Pearson correlation analysis was performed to assess collinearity among features. When the correlation coefficient between any two features exceeded 0.85, one of the features was randomly eliminated to reduce feature redundancy and mitigate the risk of model overfitting. Subsequently, mRMR and LASSO regression were sequentially applied for further feature selection and dimensionality reduction. Based on the retained features, whole-tumor radiomics scores and habitat imaging scores were calculated using a linear weighted approach. These scores were then used as independent variables to construct corresponding predictive models based on a logistic regression algorithm. Univariate and multivariate logistic regression analyses were conducted in the training cohort to identify clinical variables independently associated with PD-L1 expression status, which were subsequently used to develop the clinical prediction model. Furthermore, whole-tumor radiomics scores, habitat imaging scores, and independent clinical risk factors were integrated to establish a combined predictive model. Model discrimination was evaluated by ROC curve analysis, with sensitivity, specificity, accuracy, and AUC calculated. Decision curve analysis (DCA) was performed to assess the clinical net benefit of each model across a range of threshold probabilities. Calibration curves were used to evaluate the agreement between predicted probabilities and actual outcomes, providing a comprehensive assessment of model performance and clinical applicability.

### 2.7 Statistical analysis

All statistical analyses were performed using SPSS software (version 26.0) and R software (version 4.4.0). Continuous variables were first assessed for normality using the Kolmogorov–Smirnov test. Normally distributed variables were expressed as mean ± standard deviation and compared between groups using the independent-samples t test. Non-normally distributed variables were presented as median with interquartile range [M (Q1, Q3)] and compared using the Mann–Whitney U test. Categorical variables were expressed as counts (percentages) and compared using the chi-square test or Fisher’s exact test, as appropriate. Univariate logistic regression analysis was applied to identify clinical variables associated with PD-L1 expression status. Variables with statistical significance were subsequently entered into a multivariate logistic regression model to determine independent clinical risk factors. Differences in AUCs among predictive models were compared using the DeLong test. All statistical tests were two-sided, and a P value < 0.05 was considered statistically significant.

## 3 Results

### 3.1 Clinical characteristics and conventional CT imaging features

Comparisons of baseline clinical characteristics and conventional CT imaging features between the training and validation cohorts demonstrated no statistically significant differences in age, sex distribution, or imaging manifestations (all *P* > 0.05), indicating good comparability between the two cohorts (**Table 1**).Univariate analysis performed in the training cohort revealed that tumor location, presence of intratumoral necrosis, non-contrast CT attenuation value, and arterial-phase contrast-enhanced CT attenuation value differed significantly between patients with different PD-L1 expression levels (all *P* < 0.05; **Table 2**). In addition, patients with high PD-L1 expression showed significantly higher non-contrast and arterial-phase CT attenuation values than those with low PD-L1 expression. These variables were subsequently entered into a multivariate logistic regression model. The results showed that tumor location (OR = 2.364, 95% CI: 1.312–4.259, P = 0.004) and intratumoral necrosis (OR = 1.987, 95% CI: 1.083–3.648, P = 0.026) remained independently associated with PD-L1 expression status and were identified as independent predictors of PD-L1 expression (**Supplementary Table 2**).

**Table 1:** Comparison of baseline clinical and CT imaging characteristics between the training and validation cohorts.

Clinical variables	Training cohort (n = 172)	Validation cohort (n = 74)	χ²/Z value	*P* value
**PD-L1 expression [n (%)]**			0.124	0.725
Low expression	118 (68.6)	49 (66.2)		
High expression	54 (31.4)	25 (33.8)		
**Age (years), M (Q1, Q3)**	68 (61, 75)	67 (59, 74)	1.107	0.268
**Sex [n (%)]**			0.018	0.893
Male	131 (76.2)	56 (75.7)		
Female	41 (23.8)	18 (24.3)		
**Smoking history [n (%)]**			1.032	0.310
No	123 (71.5)	56 (75.7)		
Yes	49 (28.5)	18 (24.3)		
**Histological subtype [n (%)]**			—	0.742
Adenocarcinoma	90 (52.3)	37 (50.0)		
Squamous cell carcinoma	73 (42.4)	33 (44.6)		
Others*	9 (5.3)	4 (5.4)		
**Tumor location [n (%)]**			2.146	0.143
Upper or middle lobe	101 (58.7)	48 (64.9)		
Lower lobe	71 (41.3)	26 (35.1)		
**Lobulation [n (%)]**			0.009	0.924
Absent	47 (27.3)	21 (28.4)		
Present	125 (72.7)	53 (71.6)		
**Spiculation [n (%)]**			0.428	0.513
Absent	138 (80.2)	57 (77.0)		
Present	34 (19.8)	17 (23.0)		
**Pleural indentation [n (%)]**			0.031	0.861
Absent	64 (37.2)	28 (37.8)		
Present	108 (62.8)	46 (62.2)		
**Pleural metastasis [n (%)]**			0.364	0.546
Absent	98 (57.0)	45 (60.8)		
Present	74 (43.0)	29 (39.2)		
**Peritumoral obstructive pneumonia [n (%)]**			0.412	0.521
Absent	93 (54.1)	43 (58.1)		
Present	79 (45.9)	31 (41.9)		
**Vascular convergence [n (%)]**			2.051	0.152
Absent	76 (44.2)	25 (33.8)		
Present	96 (55.8)	49 (66.2)		
**Necrosis [n (%)]**			0.587	0.444
Absent	101 (58.7)	47 (63.5)		
Present	71 (41.3)	27 (36.5)		
**Cavitation [n (%)]**			0.083	0.773
Absent	141 (82.0)	60 (81.1)		
Present	31 (18.0)	14 (18.9)		
**Calcification [n (%)]**			1.421	0.233
Absent	132 (76.7)	61 (82.4)		
Present	40 (23.3)	13 (17.6)		
**Intrapulmonary metastasis [n (%)]**			1.086	0.297
Absent	126 (73.3)	57 (77.0)		
Present	46 (26.7)	17 (23.0)		
**Pleural or pericardial effusion [n (%)]**			0.047	0.829
Absent	112 (65.1)	49 (66.2)		
Present	60 (34.9)	25 (33.8)		
**Mediastinal or hilar lymphadenopathy [n (%)]**			0.021	0.885
Absent	58 (33.7)	26 (35.1)		
Present	114 (66.3)	48 (64.9)		
**Maximum tumor diameter (mm), M (Q1, Q3)**	46 (32, 62)	47 (33, 60)	0.612	0.540
**Non-contrast CT attenuation (HU), M (Q1, Q3)**	34 (28, 41)	35 (29, 43)	−0.598	0.550
**Arterial-phase CT attenuation (HU), M (Q1, Q3)**	60 (49, 69)	61 (50, 71)	−0.821	0.412

Bold text indicates variable names.

**Table 2:** Comparison of clinical and CT imaging characteristics between PD-L1 low-expression and high-expression groups in the training cohort.

Clinical variables	Low expression (n=118)	High expression (n=54)	χ²/Z value	P value
Age (years), M (Q1,Q3)	67 (60,74)	69 (62,76)	1.126	0.260
Male, n (%)	88 (74.6)	42 (77.8)	0.184	0.668
Smoking history, n (%)	33 (28.0)	18 (33.3)	0.512	0.474
Adenocarcinoma, n (%)	67 (56.8)	25 (46.3)	2.461	0.292
Tumor location (lower lobe), n (%)	40 (33.9)	30 (55.6)	8.325	0.004
Necrosis present, n (%)	41 (34.7)	28 (51.9)	4.936	0.026
Maximum tumor diameter (mm), M (Q1,Q3)	44 (31,59)	49 (35,65)	1.824	0.068
Non-contrast CT attenuation (HU), M (Q1,Q3)	34 (28,40)	37 (31,44)	2.148	0.032
Arterial-phase CT attenuation (HU), M (Q1,Q3)	58 (48,67)	65 (55,74)	2.965	0.003

### 3.2 Feature selection and construction of imaging scores

**Figure 2:**
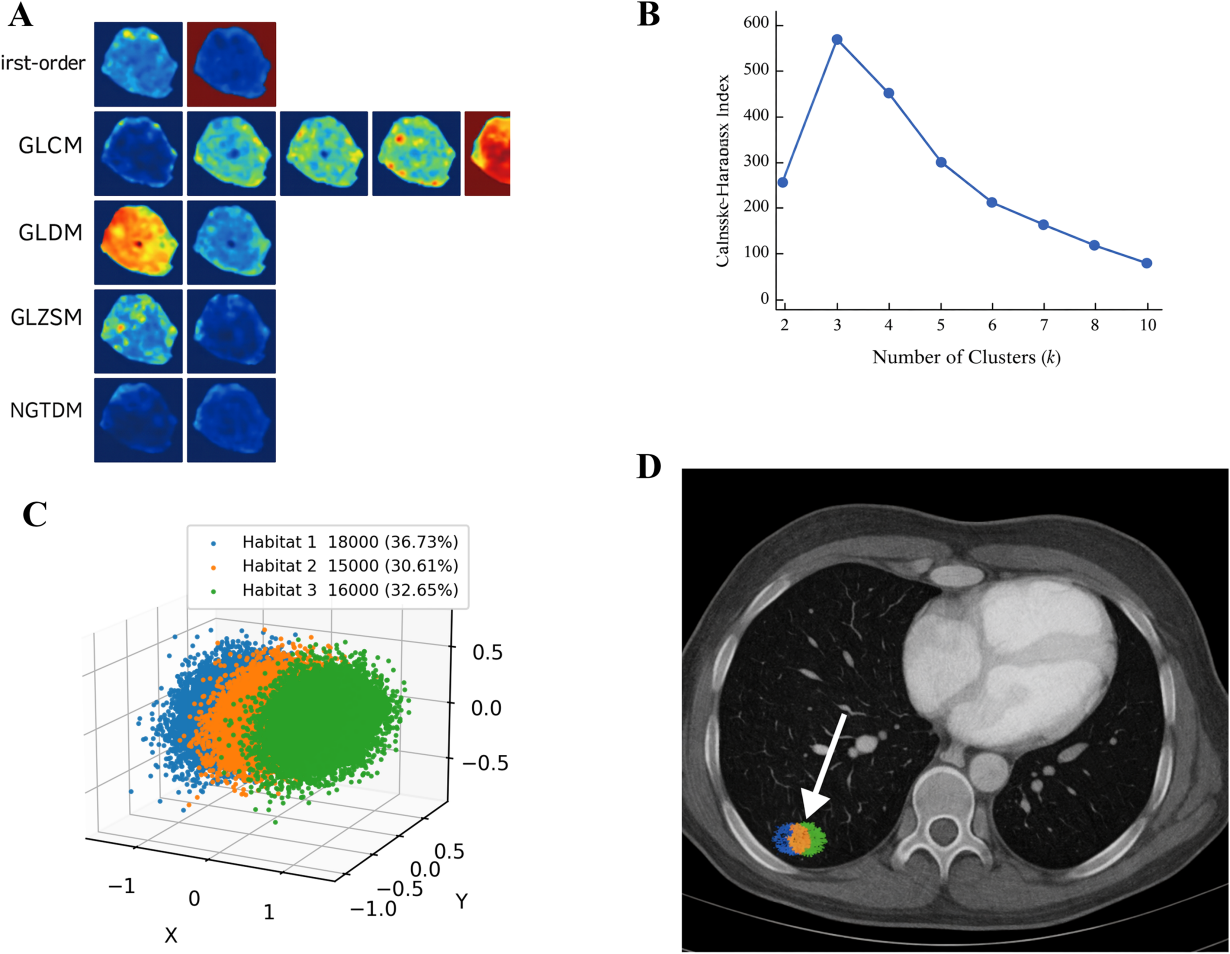
Habitat-based radiomics analysis. **(A)** Local first-order and texture radiomic feature maps. **(B)** Calinski–Harabasz index used to determine the optimal cluster number (k = 4). **(C)** Three-dimensional visualization of voxel-wise habitat clustering. **(D)** Axial CT image showing four color-coded intra-tumoral habitat subregions (arrow).

**Figure 3:**
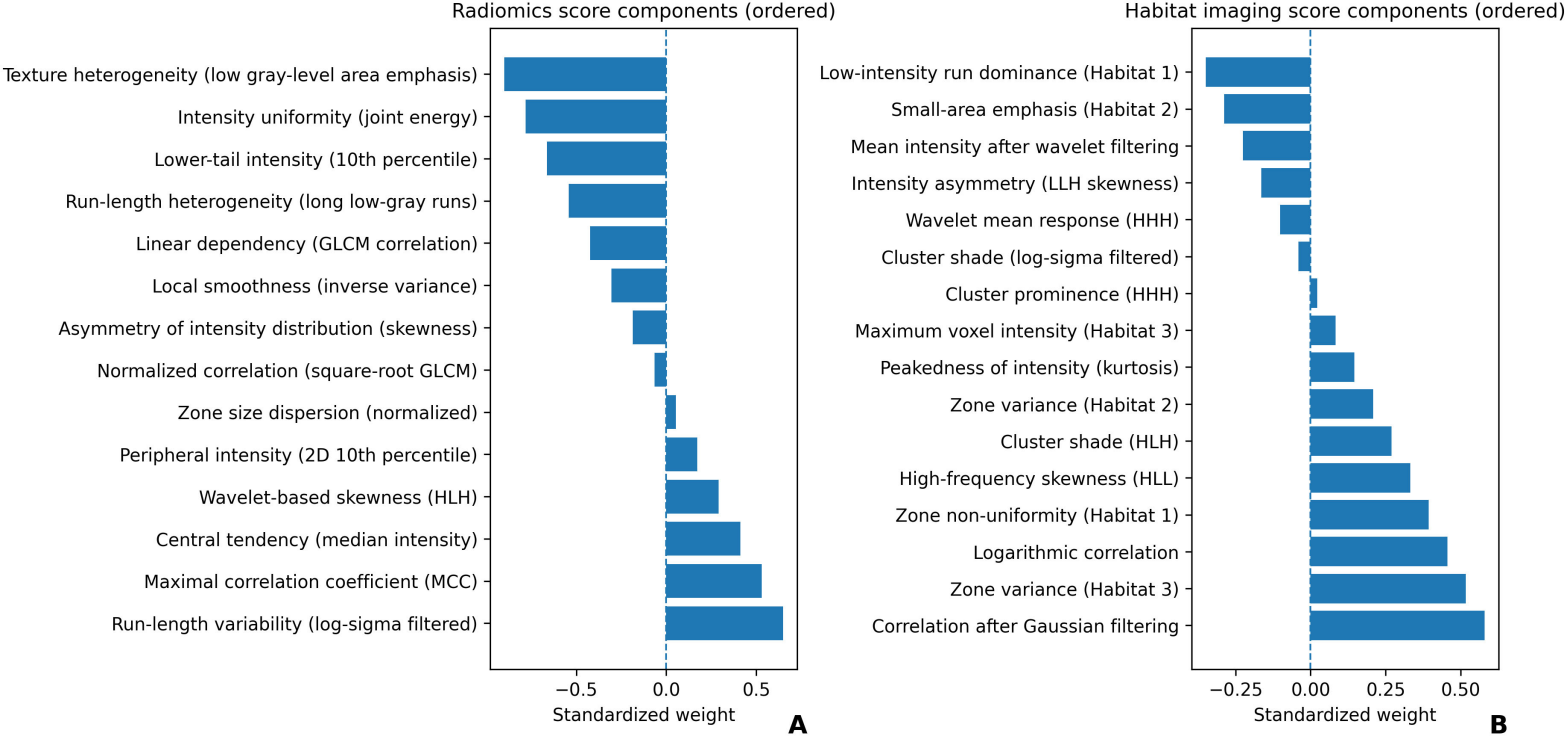
Radiomics and habitat imaging score construction. **(A)** Standardized weights of selected whole-tumor radiomic features contributing to the radiomics score (Rad-score). **(B)** Standardized weights of selected habitat imaging features contributing to the habitat imaging score (Hab-score).

In the whole-tumor radiomics analysis, a total of 1,624 radiomic features were initially extracted. After excluding duplicate and invalid features, as well as features with an ICC < 0.80, 1,372 features were retained for subsequent analysis. Based on the largest cross-sectional tumor slice, unsupervised K-means clustering was applied to the initially extracted 18 categories of local radiomic features (**Figure 2A**) to identify spatial subregions within the tumor exhibiting similar imaging characteristics. The optimal number of clusters was determined to be four by maximizing the Calinski–Harabasz index (**Figure 2B**). Voxels assigned to the same cluster were grouped into color-coded habitat subregions, resulting in four distinct intratumoral habitats (**Figures 2C, D**). Radiomic features were then extracted separately from each habitat subregion. Subregional features were subsequently integrated using a K-nearest neighbor algorithm, yielding a total of 4,986 habitat imaging features. In the training cohort, feature dimensionality reduction was sequentially performed using Pearson correlation analysis, maximum relevance minimum redundancy, and LASSO regression. Five-fold cross-validation was applied to identify the optimal feature subset, resulting in the selection of 11 whole-tumor radiomic features and 13 habitat imaging features. Based on these selected features and their corresponding regression coefficients, a whole-tumor radiomics score (Rad-score = −0.417 + Σ feature × weight) and a habitat imaging score (Hab-score = −0.268 + Σ feature × weight) were constructed, respectively (**Figure 3**).

### 3.3 Model development and performance assessment

**Figure 4:**
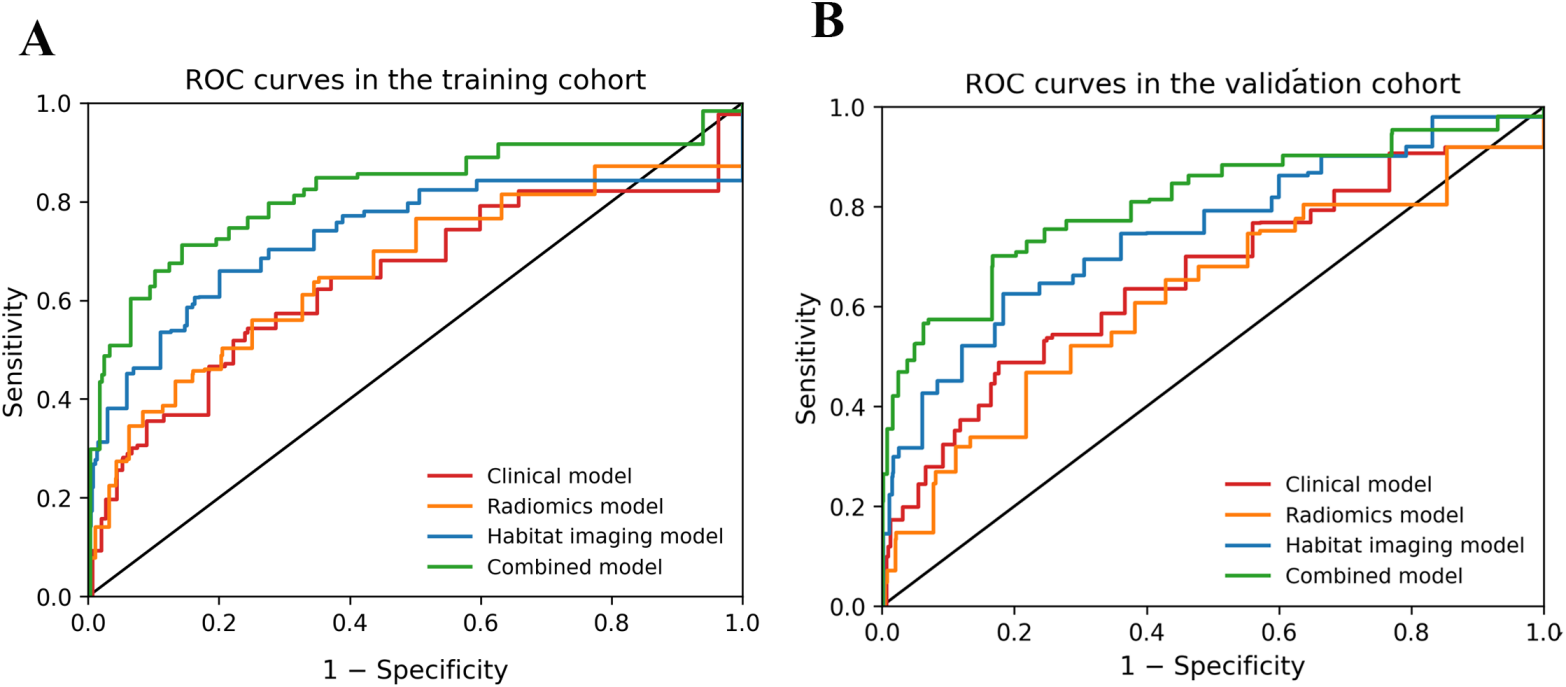
Receiver operating characteristic (ROC) curves of the predictive models. **(A)** ROC curves of the clinical, whole-tumor radiomics, habitat imaging, and combined models in the training cohort. **(B)** ROC curves of the same models in the validation cohort.

Four predictive models—including a clinical model, a whole-tumor radiomics model, a habitat imaging model, and a combined model—were developed using the whole-tumor radiomics score, habitat imaging score, and independently associated clinical risk factors. Receiver operating characteristic (ROC) curve analysis was applied to evaluate the predictive performance of each model. In the training cohort, the area under the ROC curve (AUC) values for the clinical, whole-tumor radiomics, habitat imaging, and combined models were approximately 0.683, 0.703, 0.774, and 0.840, respectively (**Figure 4A**). In the validation cohort, the corresponding AUCs were approximately 0.680, 0.649, 0.758, and 0.828, respectively (**Figure 4B**). Comparison of ROC curves demonstrated that the combined model consistently achieved the highest discriminative ability in both cohorts. DeLong’s test revealed that, in the training cohort, the combined model exhibited significantly superior discrimination compared with the whole-tumor radiomics model, habitat imaging model, and clinical model, with statistically significant differences in AUC (Z = 3.775, 2.327, and 4.015, respectively; all *P* < 0.05).

### 3.4 Calibration performance of the combined model

**Figure 5:**
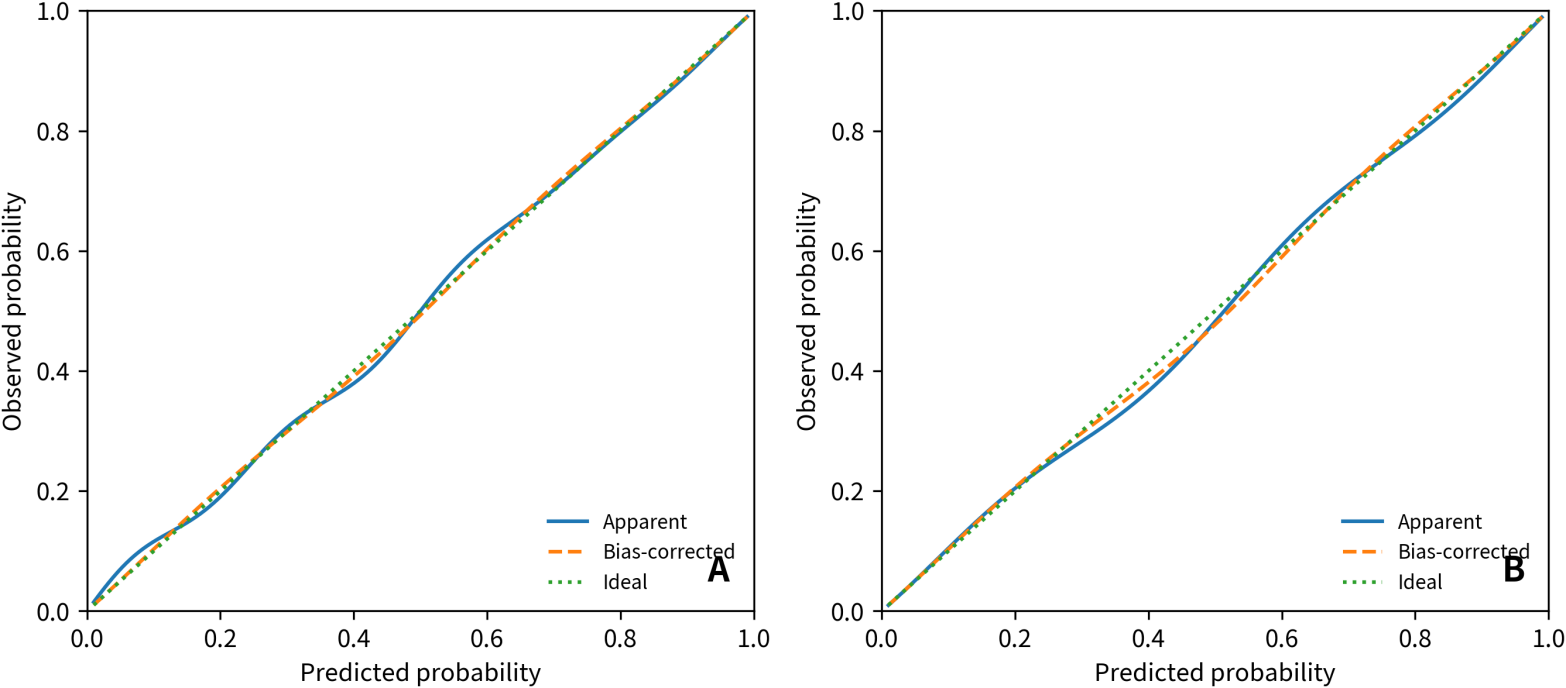
Calibration curves of the combined model for predicting PD-L1 expression in patients with NSCLC. **(A)** Calibration curve of the combined model in the training cohort. **(B)** Calibration curve of the combined model in the validation cohort. The blue solid line represents the apparent performance of the model, the orange dashed line represents the bootstrap bias-corrected performance, and the green dotted line indicates the ideal reference line.

Calibration curve analysis was performed to evaluate the agreement between the predicted probabilities and the observed outcomes of the combined model. As shown in **Figure 5**, the calibration curves demonstrated excellent consistency between predicted and actual PD-L1 expression probabilities in both the training and validation cohorts. The apparent calibration curves and bootstrap bias-corrected curves were closely aligned with the ideal reference line, indicating satisfactory calibration performance and minimal prediction bias. In addition, the Hosmer–Lemeshow goodness-of-fit test further confirmed good model calibration, with χ² = 2.048 (P = 0.359) in the training cohort and χ² = 2.346 (P = 0.309) in the validation cohort.

### 3.5 Decision curve analysis

**Figure 6:**
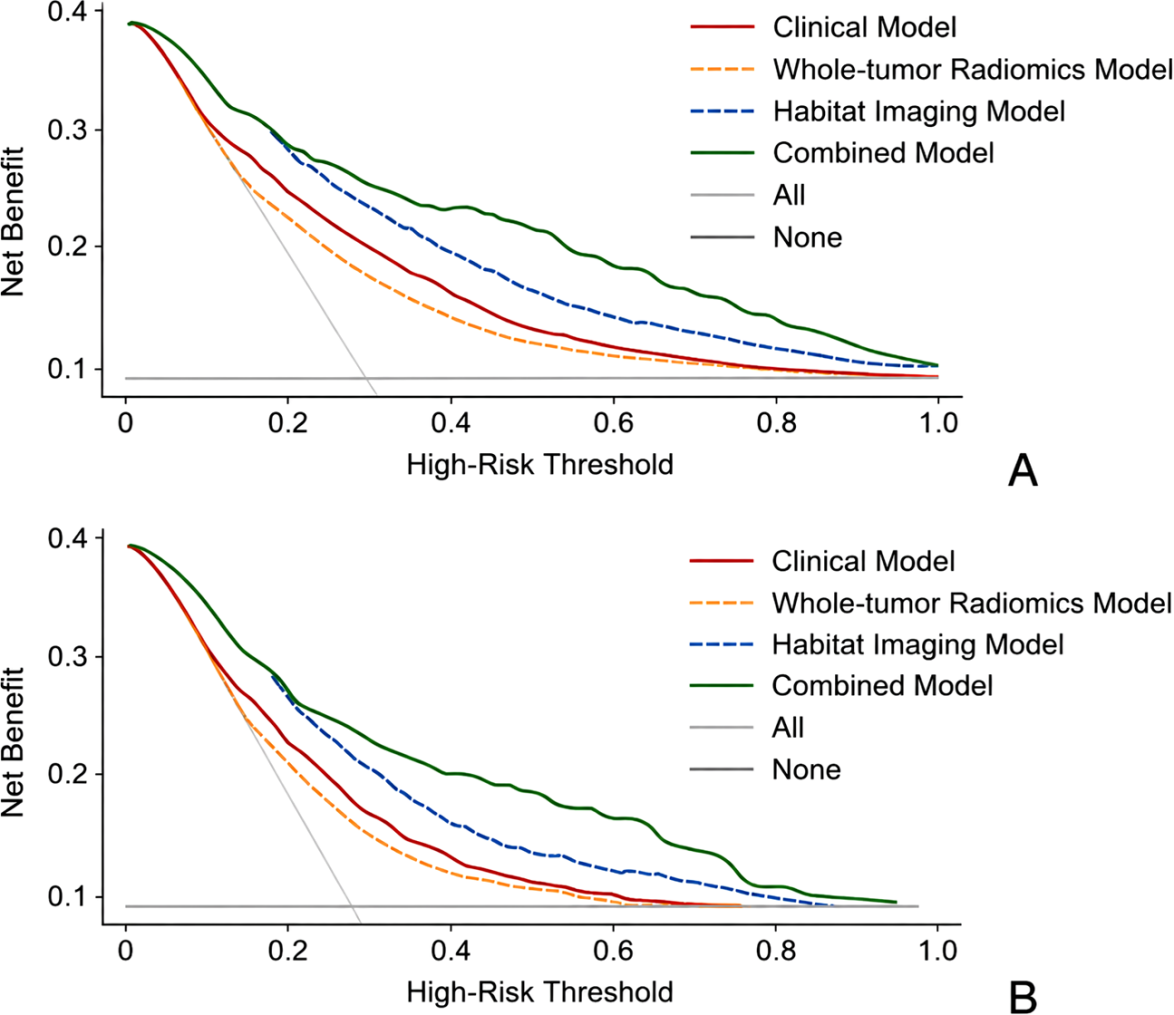
Decision curve analysis of the predictive models. **(A)** Net benefit of the clinical, omics (whole-tumor radiomics), habitat (ecological) imaging, and combined models in the training cohort across different high-risk thresholds. **(B)** Decision curve analysis of the same models in the validation cohort, showing the superior net benefit of the combined model over a wide range of threshold probabilities.

DCA was performed to evaluate the clinical utility of the clinical model, whole-tumor radiomics model, habitat imaging model, and combined model. As shown in **Figure 6**, the combined model consistently achieved the highest net benefit across a wide range of threshold probabilities in both the training and validation cohorts. Compared with the clinical model, whole-tumor radiomics model, and habitat imaging model, the combined model demonstrated superior clinical applicability and provided greater benefit for individualized prediction of PD-L1 expression. The habitat imaging model also exhibited higher net benefit than the conventional whole-tumor radiomics model over most threshold ranges. These findings indicate that integrating clinical variables, radiomics features, and habitat imaging features significantly improves the clinical usefulness of the predictive model for assessing PD-L1 expression in patients with NSCLC.

## 4 Discussion

The clinical management of NSCLC primarily relies on a multimodal strategy including surgery, radiotherapy, and chemotherapy (16).Nevertheless, substantial interpatient variability in treatment response and unsatisfactory long-term prognosis remain major clinical challenges. PD-L1, a key immune checkpoint molecule, exhibits pronounced heterogeneity in expression among patients with NSCLC (8, 17). Previous studies have demonstrated that patients with high PD-L1 expression tend to experience significantly shorter progression-free survival and overall survival compared with those with low or negative PD-L1 expression. In contrast, immune checkpoint inhibitor therapy has been shown to markedly improve survival outcomes in NSCLC patients with high PD-L1 expression (18, 19). Consequently, accurate pre-treatment assessment of PD-L1 expression status is of critical importance for individualized therapeutic planning and optimization of immunotherapy decision-making (20). From a biological perspective, PD-L1 expression is not uniformly distributed within tumors but is closely associated with multiple components of the tumor microenvironment, including hypoxia, immune cell infiltration, metabolic reprogramming, and inflammatory responses (19, 21). These processes often display substantial spatial heterogeneity within the tumor mass. Habitat imaging has emerged as a novel imaging analysis approach capable of capturing intratumoral spatial heterogeneity by partitioning tumors into subregions and integrating region-specific features (22). This noninvasive technique provides a promising avenue for linking imaging phenotypes with underlying molecular characteristics and microenvironmental complexity. Importantly, such spatial heterogeneity is increasingly recognized as a radiological surrogate of immune microenvironment diversity, which may directly influence PD-L1 expression patterns and tumor immune escape mechanisms.

In the present study, the habitat imaging–based model demonstrated superior performance in predicting PD-L1 expression status in patients with NSCLC compared with the conventional whole-tumor radiomics model, achieving relatively high AUC values in both the training and validation cohorts. Compared with earlier reports, previously published models demonstrated moderate discriminative performance in the training cohort (AUCs around 0.70), which was inferior to that observed in the present study (23). These differences suggest that traditional radiomics approaches, which typically summarize features across the entire tumor volume, may be limited in their ability to integrate complex structural and functional variations across distinct tumor regions (24). By contrast, habitat imaging explicitly decomposes tumors into spatially distinct subregions, allowing for the characterization of intra-tumoral heterogeneity arising from differences in cellular density, perfusion status, and necrotic transformation, which are all closely linked to immune regulation and PD-L1 expression. By explicitly incorporating spatial heterogeneity, habitat imaging appears to more effectively capture biologically relevant intratumoral differences associated with PD-L1 expression (25). Accumulating evidence from prior studies further supports the potential of habitat imaging in characterizing the tumor microenvironment. As an illustration, habitat imaging has been applied to quantify early therapeutic response following radiofrequency ablation of colorectal cancer lung metastases, yielding high discriminative accuracy (AUC = 0.876) and highlighting its potential role in personalized treatment planning (26). Building upon this conceptual framework, the present study further refined the methodological strategy. First, voxel-level texture features such as local entropy and energy were employed to enhance sensitivity to microscopic intratumoral heterogeneity. Second, unsupervised clustering was applied to the entire tumor region to derive biologically meaningful subregional feature maps. Such quantitative heterogeneity metrics may reflect underlying differences in tumor aggressiveness and immune microenvironment composition. This approach not only enabled intuitive visualization of spatial distributions across different feature clusters but also facilitated quantitative assessment of intratumoral heterogeneity.

The findings of the present study indicate that tumoral PD-L1 expression in patients with NSCLC is significantly associated with several clinical and imaging characteristics, among which the presence of intratumoral necrosis and tumor location within the lung were particularly prominent (27, 28). Previous studies have reported that high PD-L1 expression is frequently observed in necrotic tumor regions, suggesting a potential link between intratumoral structural alterations and immune phenotypes (29). Consistent with these reports, our results demonstrated that tumors exhibiting radiological evidence of necrosis were more likely to display elevated PD-L1 expression levels. From a biological standpoint, intratumoral necrosis often reflects rapid tumor proliferation, inadequate vascular supply, and the development of a hypoxic microenvironment. Hypoxia has been shown to upregulate PD-L1 expression through the activation of multiple signaling pathways, thereby promoting tumor immune evasion (30). This mechanistic relationship further supports the hypothesis that CT-detected necrosis can serve as a noninvasive imaging biomarker reflecting tumor immune escape and hypoxia-driven PD-L1 upregulation. This mechanistic relationship provides a plausible explanation for the observed association between imaging-detected necrosis and increased PD-L1 expression, and further supports the concept that imaging phenotypes can serve as noninvasive surrogates of underlying molecular characteristics. In addition, a significant association was observed between PD-L1 expression status and the pulmonary lobe in which the tumor was located. Anatomical and physiological differences among lung lobes, including variations in vascular perfusion, oxygen distribution, and regional microenvironmental conditions, may influence the local immune milieu and consequently modulate PD-L1 expression (31). Potentially through regional differences in ventilation–perfusion balance, immune cell distribution, and microenvironmental oxygenation. Therefore, heterogeneity in tumor location may partially account for the spatial variability of PD-L1 expression observed in NSCLC. Several limitations of this study should be acknowledged. This was a single-center retrospective analysis with a relatively limited sample size, which may restrict the generalizability of the findings. In addition, incomplete availability of actionable genomic alteration data precluded radiogenomic subgroup analysis. Moreover, peritumoral regions were not incorporated in the imaging analysis, which may limit the comprehensive assessment of tumor heterogeneity.

## 5 Conclusion

In conclusion, arterial-phase CT-based habitat imaging enables accurate and noninvasive prediction of PD-L1 expression in patients with NSCLC. The incorporation of clinical variables and whole-tumor radiomics features further enhances predictive performance. The combined model, integrating clinical, radiomics, and habitat imaging information, demonstrates superior diagnostic efficacy and provides a robust framework for pre-treatment assessment of PD-L1 status. This multidimensional approach may facilitate more precise and individualized therapeutic decision-making in NSCLC.

## Data Availability

The original contributions presented in the study are included in the article/**Supplementary Material**. Further inquiries can be directed to the corresponding author.

## References

[1] Leiter A Veluswamy RR Wisnivesky JP . The global burden of lung cancer: current status and future trends. Nat Rev Clin Oncol. (2023) 20:624–39. doi: 10.1038/s41571-023-00798-3 37479810

[2] Barta JA Powell CA Wisnivesky JP . Global epidemiology of lung cancer. Ann Glob Health. (2019) 85(1):8. doi: 10.5334/aogh.2419 30741509 PMC6724220

[3] Lin A Wei T Meng H Luo P Zhang J . Role of the dynamic tumor microenvironment in controversies regarding immune checkpoint inhibitors for the treatment of non-small cell lung cancer (NSCLC) with EGFR mutations. Mol Cancer. (2019) 18:139. doi: 10.1186/s12943-019-1062-7 31526368 PMC6745797

[4] Rahal Z El Darzi R Moghaddam SJ Cascone T Kadara H . Tumour and microenvironment crosstalk in NSCLC progression and response to therapy. Nat Rev Clin Oncol. (2025) 22:463–82. doi: 10.1038/s41571-025-01021-1 40379986 PMC12227073

[5] Khunger M Hernandez AV Pasupuleti V Rakshit S Pennell NA Stevenson J . Programmed cell death 1 (PD-1) ligand (PD-L1) expression in solid tumors as a predictive biomarker of benefit from PD-1/PD-L1 axis inhibitors: a systematic review and meta-analysis. JCO Precis Oncol. (2017) 1:1–15. doi: 10.1200/po.16.00030 35172490

[6] Incorvaia L Fanale D Badalamenti G Barraco N Bono M Corsini LR . Programmed death ligand 1 (PD-L1) as a predictive biomarker for pembrolizumab therapy in patients with advanced non-small-cell lung cancer (NSCLC). Adv Ther. (2019) 36:2600–17. doi: 10.1007/s12325-019-01057-7 31432460 PMC6822831

[7] Ilie M Hofman V Dietel M Soria JC Hofman P . Assessment of the PD-L1 status by immunohistochemistry: challenges and perspectives for therapeutic strategies in lung cancer patients. Virchows Arch. (2016) 468:511–25. doi: 10.1007/s00428-016-1910-4 26915032

[8] Matikas A Zerdes I Lövrot J Richard F Sotiriou C Bergh J . Prognostic implications of PD-L1 expression in breast cancer: systematic review and meta-analysis of immunohistochemistry and pooled analysis of transcriptomic data. Clin Cancer Res. (2019) 25:5717–26. doi: 10.1158/1078-0432.ccr-19-1131 31227501

[9] Akhtar M Rashid S Al-Bozom IA . PD-L1 immunostaining: what pathologists need to know. Diagn Pathol. (2021) 16:94. doi: 10.1186/s13000-021-01151-x 34689789 PMC8543866

[10] Han X Guo Y Ye H Chen Z Hu Q Wei X . Development of a machine learning-based radiomics signature for estimating breast cancer TME phenotypes and predicting anti-PD-1/PD-L1 immunotherapy response. Breast Cancer Res. (2024) 26:18. doi: 10.1186/s13058-024-01776-y 38287356 PMC10823720

[11] Wang C Ma J Shao J Zhang S Li J Yan J . Non-invasive measurement using deep learning algorithm based on multi-source features fusion to predict PD-L1 expression and survival in NSCLC. Front Immunol. (2022) 13:828560. doi: 10.3389/fimmu.2022.828560 35464416 PMC9022118

[12] Udayakumar D Madhuranthakam AJ Doğan BE . Magnetic resonance perfusion imaging for breast cancer. Magn Reson Imaging Clin N Am. (2024) 32:135–50. doi: 10.1016/j.mric.2023.09.012 38007276

[13] Yang X Liu M Ren Y Chen H Yu P Wang S . Using contrast-enhanced CT and non-contrast-enhanced CT to predict EGFR mutation status in NSCLC patients-a radiomics nomogram analysis. Eur Radiol. (2022) 32:2693–703. doi: 10.1007/s00330-021-08366-y 34807270 PMC8921110

[14] Ma ZP Li XL Gao K Zhang TL Wang HD Zhao YX . Application of radiomics based on chest CT-enhanced dual-phase imaging in the immunotherapy of non-small cell lung cancer. J X-Ray Sci Technol. (2023) 31:1333–40. doi: 10.3233/xst-230189 37840466

[15] Kiss FJ Járó AI Máthé D Benedek C Madaras V Manno-Kovács A . Predicting PD-L1 expression status in NSCLC using radiomic analysis of 18 F-FDG-PET/CT images. Eur J Nucl Med Mol Imaging. (2025) 53:800–11. doi: 10.1007/s00259-025-07453-2 40668270 PMC12830409

[16] Megyesfalvi Z Gay CM Popper H Pirker R Ostoros G Heeke S . Clinical insights into small cell lung cancer: tumor heterogeneity, diagnosis, therapy, and future directions. CA Cancer J Clin. (2023) 73:620–52. doi: 10.3322/caac.21785 37329269

[17] Casadevall D Clavé S Taus Á Hardy-Werbin M Rocha P Lorenzo M . Heterogeneity of tumor and immune cell PD-L1 expression and lymphocyte counts in surgical NSCLC samples. Clin Lung Cancer. (2017) 18:682–691.e5. doi: 10.1016/j.cllc.2017.04.014 28549836

[18] Ye J Ji X Dennis PA Abdullah H Mukhopadhyay P . Relationship between progression-free survival, objective response rate, and overall survival in clinical trials of PD-1/PD-L1 immune checkpoint blockade: a meta-analysis. Clin Pharmacol Ther. (2020) 108:1274–88. doi: 10.1002/cpt.1956 32564368 PMC7689755

[19] Yoon HH Jin Z Kour O Kankeu Fonkoua LA Shitara K Gibson MK . Association of PD-L1 expression and other variables with benefit from immune checkpoint inhibition in advanced gastroesophageal cancer: systematic review and meta-analysis of 17 phase 3 randomized clinical trials. JAMA Oncol. (2022) 8:1456–65. doi: 10.1001/jamaoncol.2022.3707 36006624 PMC9412834

[20] Nimmagadda S . Quantifying PD-L1 expression to monitor immune checkpoint therapy: opportunities and challenges. Cancers (Basel). (2020) 12(11):3173. doi: 10.3390/cancers12113173 33137949 PMC7692040

[21] Yi M Niu M Xu L Luo S Wu K . Regulation of PD-L1 expression in the tumor microenvironment. J Hematol Oncol. (2021) 14:10. doi: 10.1186/s13045-020-01027-5 33413496 PMC7792099

[22] Bassanelli M Sioletic S Martini M Giacinti S Viterbo A Staddon A . Heterogeneity of PD-L1 expression and relationship with biology of NSCLC. Anticancer Res. (2018) 38:3789–96. doi: 10.21873/anticanres.12662 29970498

[23] Tian P He B Mu W Liu K Liu L Zeng H . Assessing PD-L1 expression in non-small cell lung cancer and predicting responses to immune checkpoint inhibitors using deep learning on computed tomography images. Theranostics. (2021) 11:2098–107. doi: 10.7150/thno.48027 33500713 PMC7797686

[24] Moncunill V Gonzalez S Beà S Andrieux LO Salaverria I Royo C . Comprehensive characterization of complex structural variations in cancer by directly comparing genome sequence reads. Nat Biotechnol. (2014) 32:1106–12. doi: 10.1038/nbt.3027 25344728

[25] Jing SY Wang HQ Lin P Yuan J Tang ZX Li H . Quantifying and interpreting biologically meaningful spatial signatures within tumor microenvironments. NPJ Precis Oncol. (2025) 9:68. doi: 10.1038/s41698-025-00857-1 40069556 PMC11897387

[26] Huang H Chen H Zheng D Chen C Wang Y Xu L . Habitat-based radiomics analysis for evaluating immediate response in colorectal cancer lung metastases treated by radiofrequency ablation. Cancer Imaging. (2024) 24:44. doi: 10.1186/s40644-024-00692-w 38532520 PMC10964536

[27] Zheng Q Huang Y Zeng X Chen X Shao S Jin Y . Clinicopathological and molecular characteristics associated with PD-L1 expression in non-small cell lung cancer: a large-scale, multi-center, real-world study in China. J Cancer Res Clin Oncol. (2021) 147:1547–56. doi: 10.1007/s00432-020-03444-y 33196892 PMC11802070

[28] Reiniger L Téglási V Pipek O Rojkó L Glasz T Vágvölgyi A . Tumor necrosis correlates with PD-L1 and PD-1 expression in lung adenocarcinoma. Acta Oncol. (2019) 58:1087–94. doi: 10.1080/0284186x.2019.1598575 31002007

[29] Dill EA Gru AA Atkins KA Friedman LA Moore ME Bullock TN . PD-L1 expression and intratumoral heterogeneity across breast cancer subtypes and stages: an assessment of 245 primary and 40 metastatic tumors. Am J Surg Pathol. (2017) 41:334–42. doi: 10.1097/pas.0000000000000780 28195880

[30] Sun Y Tan J Miao Y Zhang Q . The role of PD-L1 in the immune dysfunction that mediates hypoxia-induced multiple organ injury. Cell Commun Signal. (2021) 19:76. doi: 10.1186/s12964-021-00742-x 34256773 PMC8276205

[31] Hsia CC Hyde DM Weibel ER . Lung structure and the intrinsic challenges of gas exchange. Compr Physiol. (2016) 6:827–95. doi: 10.1002/j.2040-4603.2016.tb00698.x 27065169 PMC5026132

