# Supplementary material for: Integrative radiomics and habitat imaging models for predicting PD-L1 expression in non-small cell lung cancer
Source: Front Oncol. 2026 Jul 6;16:1786749. doi: 10.3389/fonc.2026.1786749 (PMC13381202; doi:10.3389/fonc.2026.1786749)
Supplement: Supplementary file 1 [file Table1.docx]

| **Feature Category** | **Description** | **Number of Features*** |
| --- | --- | --- |
| Shape Features | Quantification of tumor geometry and morphology | 14 |
| First-Order Features | Distribution of voxel intensities within the ROI | 18 |
| GLCM Features | Gray-Level Co-occurrence Matrix texture features | 24 |
| GLRLM Features | Gray-Level Run Length Matrix texture features | 16 |
| GLSZM Features | Gray-Level Size Zone Matrix texture features | 16 |
| GLDM Features | Gray-Level Dependence Matrix texture features | 14 |
| NGTDM Features | Neighboring Gray Tone Difference Matrix features | 5 |
| Wavelet Features | Multi-scale texture features extracted after wavelet decomposition | 744 |
| LoG Features | Features extracted from Laplacian of Gaussian filtered images | 525 |
| Total |  | 1376 |

**Supplementary Table S1. Categories of Radiomic Features Extracted Using Py Radiomics**
