# Supplementary material for: Integrative radiomics and habitat imaging models for predicting PD-L1 expression in non-small cell lung cancer
Source: Front Oncol. 2026 Jul 6;16:1786749. doi: 10.3389/fonc.2026.1786749 (PMC13381202; doi:10.3389/fonc.2026.1786749)
Supplement: Supplementary file 2 [file Table2.docx]

**Table S2.** Multivariate Logistic Regression Analysis of Predictors of PD-L1 Expression in the Training Cohort

| **Variable** | **β coefficient** | **Standard Error** | **Wald χ²** | **OR** | **95% CI** | **P value** |
| --- | --- | --- | --- | --- | --- | --- |
| Tumor location (lower lobe vs upper/middle lobe) | 0.860 | 0.301 | 8.165 | 2.364 | 1.312–4.259 | 0.004 |
| Intratumoral necrosis (present vs. absent) | 0.687 | 0.310 | 4.909 | 1.987 | 1.083–3.648 | 0.026 |
